# Abundance of *ACVR1B* transcript is elevated during septic conditions: Perspectives obtained from a hands-on reductionist investigation

**DOI:** 10.3389/fimmu.2023.1072732

**Published:** 2023-03-20

**Authors:** Anucha Preechanukul, Thatcha Yimthin, Sarunporn Tandhavanant, Tobias Brummaier, Chalita Chomkatekaew, Sukanta Das, Basirudeen Syed Ahamed Kabeer, Mohammed Toufiq, Darawan Rinchai, T. Eoin West, Damien Chaussabel, Narisara Chantratita, Mathieu Garand

**Affiliations:** ^1^ Department of Microbiology and Immunology, Faculty of Tropical Medicine, Mahidol University, Bangkok, Thailand; ^2^ Swiss Tropical and Public Health Institute, Basel, Switzerland; ^3^ University of Basel, Basel, Switzerland; ^4^ Mahidol-Oxford Tropical Medicine Research Unit (MORU), Faculty of Tropical Medicine, Mahidol University, Bangkok, Thailand; ^5^ Department of Molecular Tropical Medicine and Genetics, Faculty of Tropical Medicine, Mahidol University, Bangkok, Thailand; ^6^ Systems Biology and Immunology Department, Sidra Medicine, Doha, Qatar; ^7^ Division of Pulmonary, Critical Care and Sleep Medicine, Department of Medicine, University of Washington, Seattle, WA, United States; ^8^ Department of Global Health, University of Washington, Seattle, WA, United States; ^9^ Division of Pediatric Cardiothoracic Surgery, Department of Surgery, Washington University School of Medicine, St. Louis, MI, United States

**Keywords:** activin A, sepsis, melioidosis, innate immunity, blood transcriptomics

## Abstract

Sepsis is a complex heterogeneous condition, and the current lack of effective risk and outcome predictors hinders the improvement of its management. Using a reductionist approach leveraging publicly available transcriptomic data, we describe a knowledge gap for the role of ACVR1B (activin A receptor type 1B) in sepsis. ACVR1B, a member of the transforming growth factor-beta (TGF-beta) superfamily, was selected based on the following: 1) induction upon *in vitro* exposure of neutrophils from healthy subjects with the serum of septic patients (GSE49755), and 2) absence or minimal overlap between ACVR1B, sepsis, inflammation, or neutrophil in published literature. Moreover, *ACVR1B* expression is upregulated in septic melioidosis, a widespread cause of fatal sepsis in the tropics. Key biological concepts extracted from a series of PubMed queries established indirect links between ACVR1B and “cancer”, “TGF-beta superfamily”, “cell proliferation”, “inhibitors of activin”, and “apoptosis”. We confirmed our observations by measuring ACVR1B transcript abundance in buffy coat samples obtained from healthy individuals (*n*=3) exposed to septic plasma (n = 26 melioidosis sepsis cases)*ex vivo*. Based on our re-investigation of publicly available transcriptomic data and newly generated *ex vivo* data, we provide perspective on the role of ACVR1B during sepsis. Additional experiments for addressing this knowledge gap are discussed.

## Introduction

Sepsis is a heterogeneous syndrome that arises from a dysregulated host inflammatory response to an infection (1–3). The response is accompanied by activation of vascular endothelial cells, neutrophils and platelets which together can contribute to collateral tissue damage in the vasculature. Inflammation worsen with the influx of neutrophils to the site of infection. Subsequent clearance of infected neutrophils are part of the way to the resolution of inflammation (4, 5). Clinical biomarkers of sepsis have been assessed (reviewed in (6)). Some, like C-reactive protein and procalcitonin, are routinely used in clinical practice but have significant limitations. The lack of clinical biomarkers for risk and outcome prediction hinders the improvement of sepsis management. As early detection and treatment of sepsis are key to favorable outcomes, predictive markers (e.g., gene expression signatures) are needed (7).

In tropical countries, melioidosis is a common cause of community-acquired infection and associated with high mortality. Melioidosis is caused by the environmental bacterium, *Burkholderia pseudomallei* via ingestion, inhalation or inoculation. The global incidence of melioidosis is estimated to be 165,000 cases with a mortality rate of approximately 89,000 cases per year (8). To improve the outcome of melioidosis patients, there is a need to understand the immunopathogenesis of severe sepsis from melioidosis.

The ACVR1B gene (*ACVR1B*) encodes the activin A receptor type 1B. Activins are a pluripotent growth and differentiation factors, believed to be involved in numerous processes such as male germ cell development (9), follicle development (10), stem cell differentiation (11) as well as immune response (12). Activin isoforms are dimeric protein complexes and belong to the transforming growth factor-beta (TGF-beta) superfamily (13). Activin A is released rapidly into the circulation during inflammation and has been shown to modulate the inflammatory response by alteration of cytokine secretion, induction of nitric oxide production, and regulation of immune cell activity (14). Activin signaling pathways involve activins binding to a heteromeric complex of receptors that consist of at least two type I and two type II receptors. Both types of receptors possess serine-threonine kinase activity and regulation of gene expression is signaled via SMAD proteins. The ACVR1B gene encodes a type I receptor which is essential for activin signaling. Mutations in this gene are associated with cancer (15), cell proliferation (16).

High-throughput profiling technologies have revolutionized biomedical research by enabling assessment of physiological as well as pathological states of biological systems at an unprecedented depth. Moreover, an increasing amount of research data is available in public repositories (e.g., NCBI Gene Expression Omnibus [GEO]). These vast data collections have been postulated to serve as valuable training materials for the next generation of biomedical data scientists (17). Here, we report the upregulation of the ACVR1B gene during sepsis in human melioidosis and discuss additional possible avenues to investigate its putative role in the pathogenesis of sepsis. We acknowledge that the definition of sepsis may vary in each study and that it is challenging to properly summarize all interpretations. Also, the pathophysiology of sepsis is known to differ between age groups, e.g., neonates vs. adults. In this study, we were interested in the host response to severe infection across lifespan, experimental settings, and cell types, therefore, we use the word sepsis or septic as a broad term to encompass this syndrome which is often appreciated as a continuum of clinical presentation.

## Materials and methods

### In silico reductionist approach

Public repositories of articles and data, such as PubMed and GEO, constitute a vast resource but they can be difficult to explore. Here we present a logical reductionist approach to investigate putative novel biomarkers for sepsis. 

The steps consist of 1) identifying a gene of interest based on its differential expression in the pathological/physiological context of interest, 2) confirming the reproducibility of the initial observation, 3) determining the current body of literature linking the gene and topic, 4) extracting the known biological concepts concerning the gene, and 5) inferring putative novel roles for the gene with literature support.

All datasets were obtained from GEO and used to confirm the initial findings in relevant clinical settings/samples. Datasets were selected without prior knowledge of ACVR1B expression levels and consisted only of human studies in which transcriptome profiles were generated in septic patients and compared to uninfected controls (**Table 1**). The task of identifying datasets was facilitated by using an interactive database recently created by our group and called SysInflam HuDB (sepsis.gxbsidra.org/dm3/geneBrowser/filteredSampleSets) (18). Other relevant information was retrieved from each GEO entry, such as: the geographic localization of the patient population, and the type of biological samples.

**Table 1:** Demographic characteristics of 26 patients with melioidosis.

Patient characteristics	
Median age in years (IQR)	58 (39-80)
Male (%)	20 (76.9)
Comorbidity (may be represented multiple times)	
Diabetes (%)	13 (50.0)
Hypertension (%)	12 (46.2)
None (%)	12 (46.2)
Clinical conditions	
Bacteremia (%) Pneumonia (%)	20 (76.9) 6 (23.1)
Fever	
< 15 days (%) ≥ 15 days (%) None (%	16 (61.5) 2 (7.7) 8 (30.8)
Clinical outcome	
Died within 28 days	13 (50.0)

### Ethical approval

The work involving human subjects was approved by the ethical committee of Faculty of Tropical Medicine, Mahidol University (approval no. MUTM 2015-002-04, MUTM 2018-046-01 and MUTM 2018-039-02), Udon Thani Hospital (approval no.6/2561), Nakhon Phanom Hospital (approval no. IEC-NKP1-No.15/2558), Roi Et Hospital (approval no. 166/2559), Buriram Hospital (approval no. BR 0032, 102.3/57), and Surin Hospital (approval no. 21/2560). This study was conducted in accordance with the principles of the Declaration of Helsinki (2008) and the International Council for Harmonization and Good Clinical Practice guidelines. Written informed consent/assent form was obtained from all participants or their legal guardians.

### Sample collection

Plasma was collected from 26 patients diagnosed with melioidosis and 9 healthy subjects. Participants were recruited at five hospitals in northeastern of Thailand (endemic region for melioidosis): Udon Thani Hospital, Nakhon Phanom Hospital, Roi Et Hospital, Buriram Hospital, and Surin Hospial. This study was part of a multi-center study of patients aged ≥15 years who were culture positive for *B. pseudomallei* from any clinical samples and admitted to the hospitals between January 2015 and December 2019. The inclusion and exclusion criteria were described previously by Kaewarpai et al. (19). Plasma was collected at the time of enrolment (within 24 hours of reporting of the culture results). Patients’ characteristics and clinical information was obtained from the medical records. Ten milliliters blood were collected from 3 healthy donors aged more than 18 years old at the Faculty of Tropical Medicine, Mahidol University, Bangkok and immediately used for cell stimulation.

### Buffy coat isolation and plasma stimulation

Buffy coat from EDTA whole blood of three healthy individuals was isolated by centrifugation at 1500 x g for 15 min. Fresh buffy coat was diluted three-fold with RPMI 1640 medium (Gibco, Invitrogen, CA, USA) supplemented with 10% fetal bovine serum (FBS, Himedia, Mumbai, India), 100 U/ml penicillin (Gibco), 100 µg/ml streptomycin (Gibco), and 2 mM GLutaMAX (Gibco) and added to 96-well plates (Costar Corporation, Cambridge, MA, USA) in duplicate wells at 2x10^5^ cells in 150 µl per well. The cells were stimulated with 50 µl of plasma from melioidosis patients (n = 26), healthy donors from an endemic area (n = 9) and autologous donors (n = 3 replicates). Stimulated buffy coat was incubated at 37^°^C in a 5% CO_2_ atmosphere incubator for 4 h.

### RNA extraction

Total RNA was extracted from buffy coat of healthy donors following stimulation using the TRIzol^®^ reagent (Thermo Fisher Scientific, Darmstadt, Germany) according to manufacturer’s instruction. The RNA was re-purified using the RNeasy Mini Kit and treated with DNase according to the manufacturer’s instruction (Qiagen, Valencia, USA). RNA quantity was measured using a nanodrop spectrophotometer (Thermo Fisher Scientific).

### Quantitative reverse-transcriptase PCR

cDNA was generated by the iScript cDNA Synthesis Kit (Bio-Rad Laboratories, Hercules, USA). Transcription level of *ACVR1B* was analyzed using real-time PCR with iTaq Universal SYBR Green (Bio-Rad). The primers for *ACVR1B* were: *ACVR1B*, forward 5′ CAGCAGAACCTTGGCGGTTTA 3′, reverse 5′ GTTGGCAGATCCCAGAGGCTAC 3′ (15) and for a housekeeping gene *HuP0* were forward 5′ GCTTCCTGGAGGGTGTCC 3′, reverse 5′ GGACTCGTTTGTACCCGTTG 3 ′ (20). The PCR reaction was conducted in the CFX96^™^ Real-Time system (Bio-Rad) as follows: 1 cycle of 95°C for 30s followed by 50 cycles of 95°C for 10s and 60°C for 30s. After amplification, melting curve analysis was carried out from 65°C to 95°C. The fold change of *ACVR1B* expression was calculated by 2^(-ΔΔCt)^, normalized with ΔCt = mean Ct of *ACVR1B* – mean Ct of *HuP0* derived from the same cDNA sample.

### Statistical analysis

The average *ACVR1B* expression was calculated from source data for case (i.e., sepsis) and control groups in each dataset. To compare case to control groups, linear fold-changes (FC) were calculated for each dataset by dividing the average expression of the cases by the average expression of the control groups. An F-test of equality of variances was used to determine whether a homoscedastic or heteroscedastic t-test was adequate to compare population means. To assess statistically significant difference in gene expression, a t-test was applied to compute a p-value. The Mann-Whitney and Kruskal-Wallis tests were used to compare differences between *ACVR1B* expression of 3 healthy donors-derived buffy coat, which was exposed to septic plasma. A of *P* < 0.05 was considered statistically significant.

## Results

### In silico reductionist approach identified *ACVR1B* as differentially expressed during sepsis

**Figure 1:**
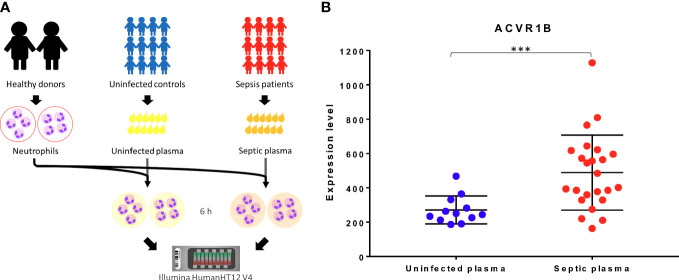
Primary observation: upregulation of ACVR1B in neutrophils in response to septic plasma. **(A)** The study design of dataset. The study was conducted in adult patients with sepsis in Khon Kaen province, northeast of Thailand. Polymorphonuclear cells (PMN) were isolated from two healthy donors. Each isolation of PMN was exposed to 20% of heparinized plasma samples which were collected from uninfected patients (n = 12) and patients with culture-confirmed sepsis (n = 12) for 6 h. The cultures in the presence of medium alone or lipopolysaccharide were used as controls (not shown). The RNA expression profile was determined using Illumina HumanHT12 V4.0 BeadChips. The dataset was deposited in the NCBI GEO public repository (GSE49755) **(B)** *ACVR1B* expression in neutrophils after exposure to uninfected plasma and septic plasma for 6 h was shown in the graph. The y-axis denotes the quantile normalized intensity values of the probes. The error bar demonstrated the mean ± standard deviation. Significant differences were determined by using the following statistical tests: T-test and F-test which results in p<0.005 (***) and p<0.001, respectively.

The *ACVR1B* gene was identified from data we previously generated (GSE49755) (21). An increase in ACRV1B transcript abundance was observed upon the exposure of neutrophils from healthy donors to plasma of septic patients. Briefly in this study, polymorphonuclear neutrophils (PMNs) were obtained from two healthy donors, isolated, and incubated for 6 hours with the plasma from 12 patients with confirmed melioid sepsis and 12 uninfected controls, respectively. PMN transcriptional profiles were generated using Illumina Bead arrays. As shown in **Figure 1A**, *ACVR1B* gene expression levels and variance between exposure to plasma from the control and septic groups were significantly different (**Figure 1B**; Additional File 1).

The current body of literature on the overlap of ACVR1B on one hand and sepsis, inflammation, or neutrophil on the other hand was assessed. Literature was retrieved using a PubMed query which comprised the official gene symbol, name and known aliases for ACVR1B, with the search restricted to the title and abstract: ACVR1B[tw] OR ACTRIB[tw] OR ALK4[tw] OR SKR2[tw] OR Actr-IB[tw] OR ALK-4[tw]. As of August of 2022, this query returned 510 results. In a Boolean search, no overlap was found between the ACVR1B literature and the search term “sepsis”. Extending the search to the literature on inflammation and neutrophil returned 13 and 2 articles, respectively. **Supplementary Figure 1** summarizes the number of articles returned in the respective searches.

### The increase in *ACVR1B* transcript abundance in the context of sepsis was validated in independent datasets

**Figure 2:**
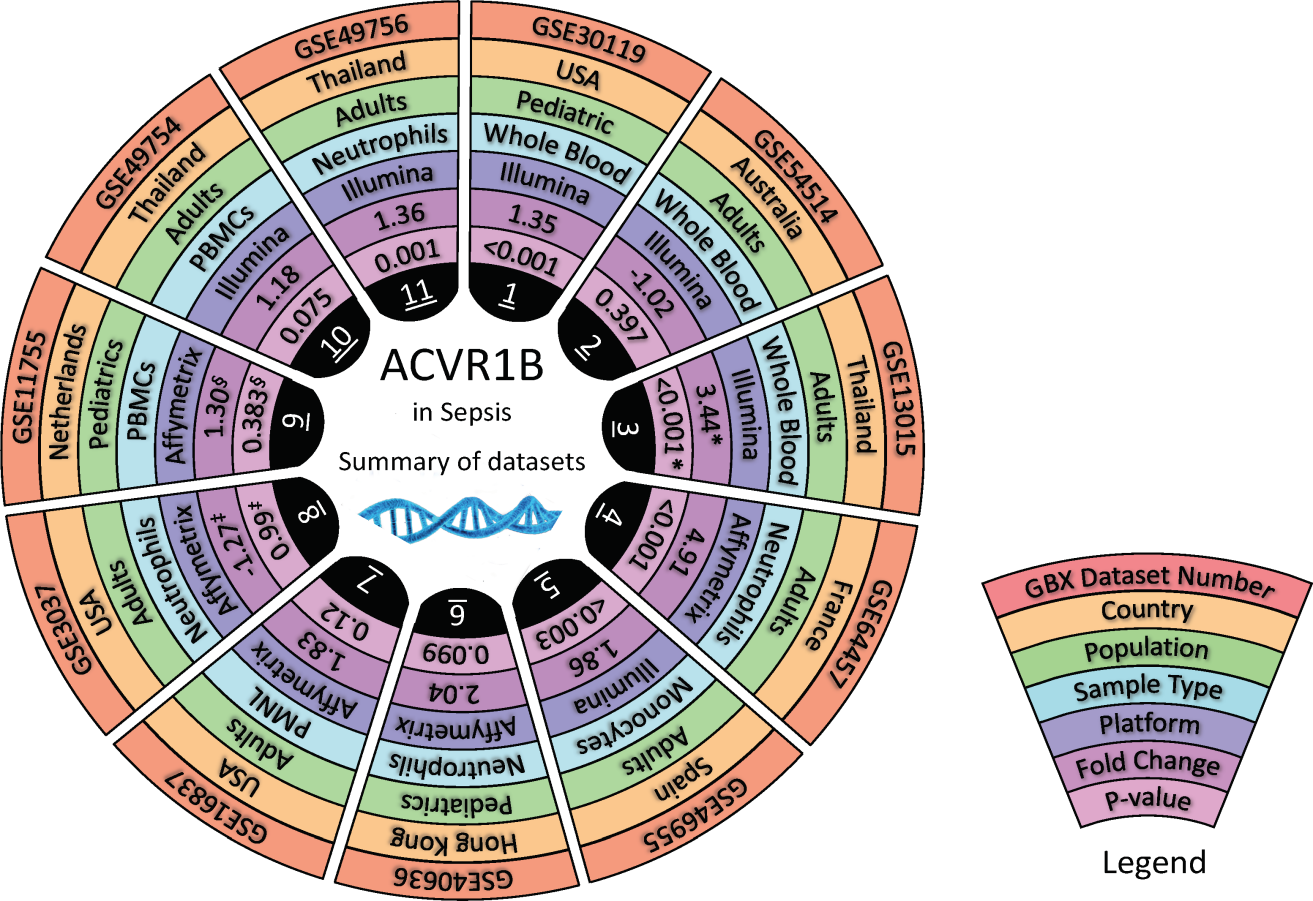
*In silico* validation of *ACVR1B* gene expression in sepsis. Characteristics of 11 datasets are shown in a concentric circle chart with each segment representing one dataset. From the outside to the inside, the following information is depicted: Gene Expression Omnibus (GEO) identification number, country in which samples were acquired, study population, sample type, and analysis platform. The inner 2 circles represent the fold-change (FC) and the p-value for comparing samples from septic patients to healthy controls. Average *ACVR1B* expression was calculated from source data for sepsis and control groups in each dataset and used to calculate FC. * Dataset contains gene expression data of healthy individuals as well as patients with sepsis caused by (*B*) *pseudomallei* and sepsis caused by other pathogens. Two comparisons were performed for this dataset. However, FC and p-value are only shown for patients with sepsis caused by (*B*) *pseudomallei* and compared to healthy controls. ^‡^ In this study, gene expression from neutrophils harvested from patients with sepsis-induced acute lung injury and stimulated with *High Mobility Group Box Protein 1* (HMGB1) as well as lipopolysaccharides were compared to neutrophils from healthy controls stimulated in the same manner. Fc and p-value are only shown for neutrophil stimulation with lipopolysaccharides at 1 hour after incubation. ^§^ In this dataset gene expression of monocytes and lymphocytes were compared between healthy controls and patients with meningococcal meningitis 24 hours after admission to the pediatric intensive care unit. Fc and p-value are only shown for *AVCR1B* expression in monocytes.

Datasets relevant to septic conditions (clinically or experimentally) were retrieved from GEO, made available on Gene Expression Browser (GXB) GXB, and used for independent validation of our initial finding. The characteristic of the datasets retrieved are shown in **Figure 2** and **Supplementary Table 1**. These datasets encompassed gene expression profiling of *in-vivo, ex-vivo* and *in-vitro* experiments, and were all derived from human samples. Various inflammatory conditions, including cell exposure to bacterial products, were evaluated in case-control designs. Strong evidence (p < 0.05) for an increased *ACVR1B* expression in sepsis was found in 6 out of 11 datasets. The other five datasets had a p-value between 0.1 and 0.05. Four of those datasets were from experiments performed *in-vitro.* These four *in vitro* experiments all have smaller sample size (ranging from 3 to 17) when compared to the *in-vivo* experiments (ranging from 13 to 127). Te magnitude of *ACVR1B* expression was associated with the experimental conditions, such as the cell types used and whether plasma of septic patients or synthetic bacterial components (e.g., bacterial lipopolysaccharide) was used.

### Regulation of cell survival and proliferation indirectly links ACVR1B with sepsis

As a first step to bridging the gap between the postulated upregulation of ACVR1B in sepsis and the rational for this observation, another systematic literature search was conducted in PubMed. The following query, restricted to title-words and excluding the term “homeobox” to avoid irrelevant gene target (ALK4 can also referred to Aristaless-like homeobox 4), was executed: ACVR1B[ti] OR ACTRIB[ti] OR ALK4[ti] OR ALK-4[ti] NOT (“genes, homeobox”[MeSH Terms] OR (“genes”[All Fields] AND “homeobox”[All Fields]) OR “homeobox genes”[All Fields] OR “homeobox”[All Fields]) AND “humans”[MeSH Terms].

We obtained 33 articles and scanned the titles to extract key biological processes/terms and fit them into 5 biologically relevant categories: biological process, biomolecules, pathway, disease, and cell type. A word-cloud summary of the words contained in the titles is shown **Supplementary Figure 1B**. Next, literature searches were performed on the intersection between ACVR1B and each of these key biological processes/terms. To maximize the output, the search queries regarding ACVR1B, and its synonyms were extended to text words ([tw]) as follows: ACVR1B[tw] OR ACTRIB[tw] OR ALK4[tw] OR SKR2[tw] OR Actr-IB[tw] OR ALK-4[tw]. A summary of the categories and concepts is shown in **Supplementary Figure 1C**.

### *Ex vivo* validation of *ACVR1B* gene expression in buffy coat exposed to septic plasma

**Figure 3:**
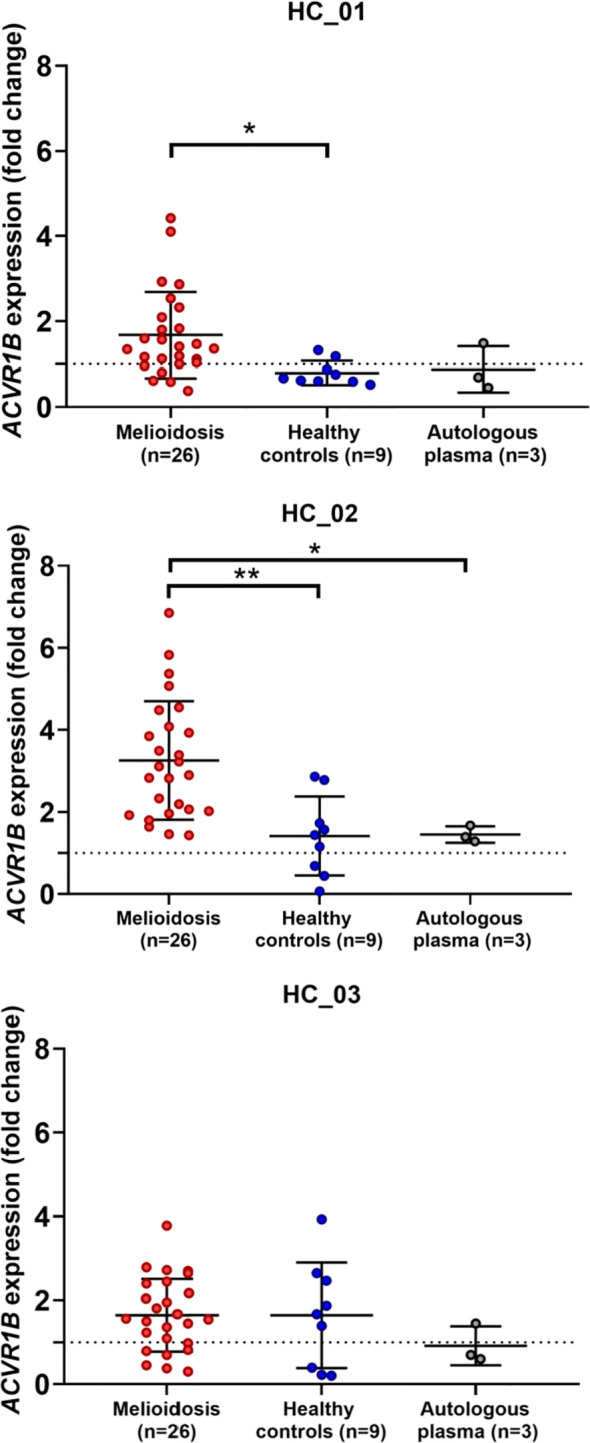
*Ex vivo* validation of *ACVR1B* upregulation in buffy coat in response to plasma from septic melioidosis patients. Buffy coat was isolated from heparinized whole blood of three healthy individuals (HC_01, HC_02 and HC_03). Each isolation of buffy coat was exposed to 25% plasma obtained from melioidosis patients (n = 26), healthy control in endemic area (n = 9) and autologous plasma (n = 3, i.e., replicates). *ACVR1B* gene expression of stimulated buffy coat were determined by RT-qPCR. Fold change in ACVR1B gene expression represent the response to exposure to melioidosis, healthy control, and autologous plasma. Scatter plots of *ACVR1B* expression show fold changes of buffy coat exposed to different plasma samples. Shown are the medians with interquartile ranges and each dot represents data obtained from one individual sample. A Kruskal-Wallis test was performed for comparison and two-tailed *P* values were calculated. Comparision with statistically significant differences are indicated (*p <0.05; **p <0.01).

To test the findings of our in silico exploration, we measured *ACVR1B* transcript abundance in the buffy coat from healthy individuals that has been incubated with septic plasma. Septic plasma obtained from melioidosis patients were used for *ex vivo* validation. The clinical characteristics of melioidosis patients are shown in **Table 1**. Buffy coats were isolated from three healthy donors and stimulated for 4 hours with plasma from melioidosis patients (n=26) and healthy controls from endemic areas (n=9), and autologous plasma (n=3). *ACVR1B* expression was measured by RT-qPCR. Abundance of *ACVR1B* transcripts was significantly higher in cells from two healthy donors (HC-01 and HC-02) stimulated with plasma of melioidosis patients compared with plasma of healthy donors (*p* < 0.01) (**Figure 3**).

### *ACVR1B* gene expression in buffy coat exposed to plasma and characteristics of melioidosis patients

As melioidosis is associated with many underlying diseases, the patients’ preexisting conditions/status and potential consequences (i.e., bacteremia, pneumonia) of an infection with *B. pseudomallei* were examined as factors of *ACVR1B* gene expression in buffy coat after exposure to plasma of melioidosis patients. Blood cells of 3 healthy donors were incubated with plasma from patients with melioidosis. *ACVR1B* expression was significantly different between survivors and non-survivors (p<0.05), but no differences were found between male and female, diabetes and non-diabetes, bacteraemia and non-bacteremia, hypertension and non-hypertension, pneumonia, and non-pneumonia (**Supplementary Figure 2**).

## Discussion

High-throughput profiling technologies have changed our understanding of biological systems. More and more data are shared in public repositories, paving new avenues for exploration of these datasets from various angles. Following this approach, we described how we identified and validated the changes in *ACVR1B* gene expression in septic melioidosis *in silico* and *ex vivo*.

We provided evidence that the ACVR1B gene is upregulated during sepsis, a finding that is substantiated by its known physiological role (e.g., in cell proliferation and apoptosis). To our knowledge there is no previous assessment of ACVR1B in melioidosis. Although induction of *ACVR1B* transcription was observed in several cohort studies assessing adult and pediatric sepsis, additional experimental data are necessary to confirm the role of ACVR1B in sepsis. Validation of our preliminary findings could be achieved by an *in vitro* culture system by exposing neutrophils to septic plasma from patients as it was done in the initial study described in **Figure 1**. Furthermore, changes in *ACVR1B* gene expression can be confirmed through qPCR in healthy whole blood, leukocytes and neutrophils (negative selection purification) upon exposure to septic plasma. Knowing that not all mRNA transcripts are translated and resulting in protein formation, a next step would be to verify whether an increase in abundance observed at the transcript level translates in increase in protein abundance. 

We observed variable responses from HC-03 to septic plasma which suggest other physiological factors influenced the host production of *ACVR1B*. Our healthy donors satisfied the inclusion/exclusion criteria (**Supplementary Table 2**) and had similar age. There is no evidence that would lead us to think that sex and blood type can affect *ACVR1B* expression in response to septic plasma. Of note, however, single nucleotide polymorphisms (SNP) in the ACVR1B gene have been associated with increased risk of lung cancer from tobacco smoke exposure (22). Another more recent study found possible implication for ACVR1B gene SNPs in chronic obstructive pulmonary disease (23). Based on these observations linking ACVR1B and inflammatory conditions, we speculate that *ACVR1B* polymorphisms may be one of the factors influencing gene expression in the HC-03 individual. While we didn’t look at gene polymorphisms of the healthy donors in this study, the subject will remain a point of interest for future work.

Additional inferences can be made by exploring the literature on ACVR1B. For example, during the latency period in human cytomegalovirus infections, miR-UL148D is one of the most expressed micro-RNAs (miRNA) in myeloid cells and directly targets ACVR1B (16). This result could suggest that ACVR1B is the target of an immune evasion strategy employed by the virus. It could be of interest to measure the co-expression of miRNAs during sepsis and assess if, in particular, miR-UL148D correlates with *ACVR1B* gene expression and the severity of symptoms. Interestingly, another miRNA, miR-199a-5p, has been shown to inhibit monocyte/macrophage differentiation by targeting ACVR1B (24); additional downstream experiments could aim to associate miR-199-5p expression with the immune responses from monocyte/macrophages. Finally, other mechanisms for the increased expression of *ACVR1B* may involve the regulation of apoptosis in leukocytes. Apoptosis in immune cells, such as neutrophils, can help eliminate infected cells, thus reducing the pathogen burden.

## Data availability statement

The raw data supporting the conclusions of this article will be made available by the authors, without undue reservation.

## Ethics statement

The studies involving human participants were reviewed and approved by Faculty of Tropical Medicine, Mahidol University, Udon Thani Hospital, Nakhon Phanom Hospital, Roi Et Hospital, Buriram Hospital, and Surin Hospital. The patients/participants provided their written informed consent to participate in this study.

## Author contributions

Conceptualization: MG, NC, DR, DC. Data curation and validation: MG, AP, TY, NC, TB, CC, ST, SD. Visualization: MG, TB, CC, ST, SD, NC, TEW Analysis and interpretation: MG, AP, TY, TB, CC, ST, SD, NC. Writing of the first draft: MG, AP, TY, TB, CC, ST, SD. Funding acquisition: DC, TEW, and NC. Methodology development: MG, DC, DR, AP, TY, NC. Project administration: MG, NC and DC. Software development: MT. Writing – review & editing MG, NC, TEW, AP, TY, TB, CC, ST, SD, DR, BS, MT, DC. The contributor’s roles listed above follow the Contributor Roles Taxonomy (CRediT) managed by The Consortia Advancing Standards in Research Administration Information (CASRAI) (https://casrai.org/credit/). All authors contributed to the article and approved the submitted version.

## References

[1] van der Poll T van de Veerdonk FL Scicluna BP Netea MG. The immunopathology of sepsis and potential therapeutic targets. Nat Rev Immunol (2017) 17(7):407–20. doi: 10.1038/nri.2017.36 28436424

[2] Rubio I Osuchowski MF Shankar-Hari M Skirecki T Winkler MS Lachmann G. Current gaps in sepsis immunology: New opportunities for translational research. Lancet Infect Dis (2019) 19(12):e422–36. doi: 10.1016/S1473-3099(19)30567-5 31630991

[3] Oikonomakou M Z Gkentzi D Gogos C Akinosoglou K. Biomarkers in pediatric sepsis: A review of recent literature. biomark Med (2020) 14(10):895–917. doi: 10.2217/bmm-2020-0016 32808806

[4] Greenlee-Wacker MC. Clearance of apoptotic neutrophils and resolution of inflammation. Immunol Rev (2016) 273(1):357–70. doi: 10.1111/imr.12453 PMC5000862 27558346

[5] Rosales C. Neutrophil: A cell with many roles in inflammation or several cell types? Front Physiol (2018) 9:113. doi: 10.3389/fphys.2018.00113 29515456 PMC5826082

[6] Pierrakos C Vincent JL. Sepsis biomarkers: A review. Crit Care (2010) 14(1):R15. doi: 10.1186/cc8872 20144219 PMC2875530

[7] Lukaszewski RA Yates AM Jackson MC Swingler K Scherer JM Simpson AJ. Presymptomatic prediction of sepsis in intensive care unit patients. Clin Vaccine Immunol (2008) 15(7):1089–94. doi: 10.1128/CVI.00486-07 PMC2446638 18480235

[8] Limmathurotsakul D Golding N Dance DAB Messina JP Pigott DM Moyes CL. Predicted global distribution of burkholderia pseudomallei and burden of melioidosis. Nat Microbiol (2016) 1:15008. doi: 10.1038/nmicrobiol.2015.8 27571754

[9] Miles DC Wakeling SI Stringer JM van den Bergen JA Wilhelm D Sinclair AH. Signaling through the TGF beta-activin receptors ALK4/5/7 regulates testis formation and male germ cell development. PloS One (2013) 8(1):e54606. doi: 10.1371/journal.pone.0054606 23342175 PMC3546992

[10] Li R Phillips DM Mather JP. Activin promotes ovarian follicle development *in vitro* . Endocrinology (1995) 136(3):849–56. doi: 10.1210/endo.136.3.7867593 7867593

[11] Sulzbacher S Schroeder IS Truong TT Wobus AM. Activin a-induced differentiation of embryonic stem cells into endoderm and pancreatic progenitors-the influence of differentiation factors and culture conditions. Stem Cell Rev (2009) 5(2):159–73. doi: 10.1007/s12015-009-9061-5 19263252

[12] Qi Y Ge J Ma C Wu N Cui X Liu Z. Activin a regulates activation of mouse neutrophils by Smad3 signalling. Open Biol (2017) 7(5):1–10. doi: 10.1098/rsob.160342 PMC5451541 28515224

[13] Xia Y Schneyer AL. The biology of activin: Recent advances in structure, regulation and function. J Endocrinol (2009) 202(1):1–12. doi: 10.1677/JOE-08-0549 19273500 PMC2704481

[14] Phillips DJ de Kretser DM Hedger MP. Activin and related proteins in inflammation: not just interested bystanders. Cytokine Growth Factor Rev (2009) 20(2):153–64. doi: 10.1016/j.cytogfr.2009.02.007 19261538

[15] Togashi Y Sakamoto H Hayashi H Terashima M de Velasco MA Fujita Y. Homozygous deletion of the activin a receptor, type IB gene is associated with an aggressive cancer phenotype in pancreatic cancer. Mol Cancer (2014) 13:126. doi: 10.1186/1476-4598-13-126 24886203 PMC4047430

[16] Lau B Poole E Krishna B Sellart I Wills MR Murphy E. The expression of human cytomegalovirus MicroRNA MiR-UL148D during latent infection in primary myeloid cells inhibits activin a-triggered secretion of IL-6. Sci Rep (2016) 05:6:31205. doi: 10.1038/srep31205 PMC4974560 27491954

[17] Chaussabel D Rinchai D. Using “collective omics data” for biomedical research training. Immunology. (2018) 155(1):18–23. doi: 10.1111/imm.12944 29705995 PMC6099165

[18] Toufiq M Huang SSY Boughorbel S Alfaki M Rinchai D Saraiva LR. SysInflam HuDB, a Web Resource for Mining Human Blood Cells Transcriptomic Data Associated with Systemic Inflammatory Responses to Sepsis. J Immunol. (2021) 207(9):2195–202. 10.4049/jimmunol.2100697 PMC8525868 34663591

[19] Kaewarpai T Ekchariyawat P Phunpang R Wright SW Dulsuk A Moonmueangsan B. Longitudinal profiling of plasma cytokines in melioidosis and their association with mortality: A prospective cohort study. Clin Microbiol Infect (2020) 26(6):783.e1–8. doi: 10.1016/j.cmi.2019.10.032 PMC7647866 31705997

[20] Cliff JM Cho JE Lee JS Ronacher K King EC van Helden P. Excessive cytolytic responses predict tuberculosis relapse after apparently successful treatment. J Infect Dis (2016) 213(3):485–95. doi: 10.1093/infdis/jiv447 PMC4704670 26351358

[21] Khaenam P Rinchai D Altman MC Chiche L Buddhisa S Kewcharoenwong C. A transcriptomic reporter assay employing neutrophils to measure immunogenic activity of septic patients’ plasma. J Transl Med (2014) 12:65. doi: 10.1186/1479-5876-12-65 24612859 PMC4007645

[22] Spitz MR Gorlov IP Amos CI Dong Q Chen W Etzel CJ. Variants in inflammation genes are implicated in risk of lung cancer in never smokers exposed to second-hand smoke. Cancer Discovery (2011) 1(5):420–9. doi: 10.1158/2159-8290.CD-11-0080 PMC3919666 22586632

[23] Morrow JD Cho MH Platig J Zhou X DeMeo DL Qiu W. Ensemble genomic analysis in human lung tissue identifies novel genes for chronic obstructive pulmonary disease. Hum Genomics (2018) 12(1):1. doi: 10.1186/s40246-018-0132-z 29335020 PMC5769240

[24] Lin HS Gong JN Su R Chen MT Song L Shen C. miR-199a-5p inhibits monocyte/macrophage differentiation by targeting the activin a type 1B receptor gene and finally reducing C/EBPα expression. J Leukoc Biol (2014) 96(6):1023–35. doi: 10.1189/jlb.1A0514-240R 25258381

