# Supplementary material for: Abundance of ACVR1B transcript is elevated during septic conditions: Perspectives obtained from a hands-on reductionist investigation
Source: Front Immunol. 2023 Mar 20;14:1072732. doi: 10.3389/fimmu.2023.1072732 (PMC10067751; doi:10.3389/fimmu.2023.1072732)
Supplement: Supplementary file 1 [file DataSheet_1.pdf]

SI Figure 1

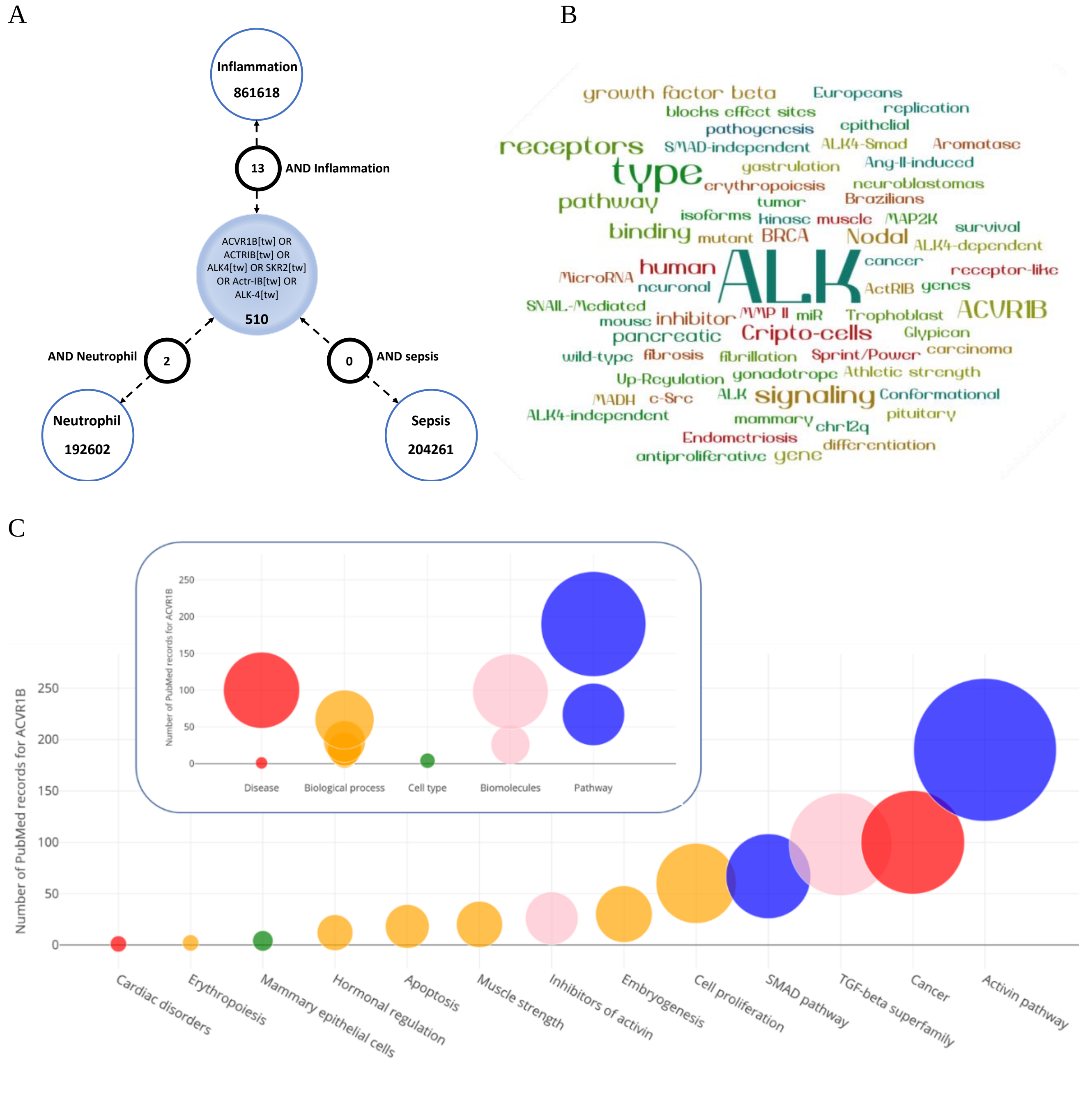

Supplementary Fig 1. Results of literature search for the gene *ACVR1B* (and alias) and sepsis, inflammation, and neutrophils in human. **A)** The number in the circles represents the frequency of articles retrieved for each search. The intersections between ACVR1B and each term are represented by a dashed line. **B)** Word cloud depicting the frequency of the terms from the 33 articles obtained from a PubMed query, restricted to article title only, about ACVR1B and its alias; note that this includes the gene aliases ALK4 or ALK-4. **C)** Categories and processes covering the key concepts extracted from title-restricted literature mining. Literature searches were performed on the intersection between ACVR1B and each of these key biological processes/terms. The size of the circles is proportional to the number of PubMed records retrieved and color is coded the biologically relevant categories. The inset plot summarizes the results into the 5 categories.

SI Table 1. Characteristics of the datasets used for validation of the initial observation.

Datasets	Title	Group A (control)	Group B (sepsis)	Population group	Cell types	Number of sample			Expression group A	Expression group B	B/A ratio	t-test *	F-test	FC (from GXB)	Platform	Country
						Total	B	A								
In vivo																
GSE30119	Genome-wide analysis of whole blood transcriptional response to community-acquired Staphylococcus aureus infection in vivo	Healthy individuals	Patients with S. aureus infection	Pediatrics	Whole blood	143	99	44	235.846	317.438	1.35	6.55E-08	5.69E-07	1.35	Illumina	USA
GSE54514	Whole blood transcriptome of survivors and nonsurvivors of sepsis	Healthy individuals	Patients with sepsis (survivors)	Adults	Whole blood	163	127	36	350.16	333.12	-1.02	3.97E-01	7.05E-02	-1.02	Illumina	Australia
GSE13015	Genomic Transcriptional Profiling Identifies a Blood Biomarker Signature for the Diagnosis of Septicemic Melioidosis-GSE13015-Healthy-Melioidosis-Other Sepsis-T2D	Healthy individuals	Patients with sepsis caused by B. Pseudomallei	Adults	Whole blood	21	16	5	220.78	736.44	3.34	2.81E-07	2.67E-04	3.34	Illumina	Thailand
		Healthy individuals	Patients with sepsis caused by other pathogens	Adults		18	13	5	220.78	622.92	2.82	3.98E-05	2.99E-04	2.82		
Ex vivo																
GSE64457	Marked alterations of neutrophil functions during sepsis-induced immunosuppression	Healthy individuals	Patients with septic shoc with features of sepsis-induced immunosuppression	Adults	Neutrophils	23	15	8	67.37	330.97	4.91	7.80E-05	2.15E-07	4.9	Affymetrix	France
In vitro																
GSE46955	Transcriptome analysis of blood monocytes from sepsis patients	Healthy individuals	Patients with sepsis	Adults	Monocytes	14	8	6	297.09	553.93	1.86	3.35E-03	1.37E-02	1.87	Illumina	Spain
GSE40636	PGN induced transcriptional changes in human neonatal neutrophils	Unstimulated (4 hrs)	Peptidoglycan stimulation (4 hrs)	Pediatrics	Neutrophils from cord blood	6	3	3	51.04	104.38	2.04	9.89E-02	3.57E-03	2.04	Affymetrix	Hongkong
GSE16837	Gene expression data from S. aureus-exposed neutrophils	Unstimulated (3 hrs)	S. aureus stimulation (3 hrs, strain 9897)	Adults	Polymorphonuclear leukocytes	8	4	4	1393.3	2553.3	1.83	1.20E-02	3.73E-01	1.83	Affymetrix	USA
GSE3037	Stimulation by LPS and HMGB1 in peripheral blood neutrophils from patients with sepsis-induced acute lung injury	Unstimulated (1 hr)	HMGB1 stimulation (1 hr)	Adults	Neutrophils	16	8	8	6.76	8.26	-1.21	4.32E-01	4.78E-02	-1.21	Affymetrix	USA
		Unstimulated (1 hr)	LPS stimulation (1 hr)	Adults		16	8	8	6.76	6.75	-1.27	9.90E-01	4.63E-01	-1.27		
GSE11755	Gene expression profiling in pediatric meningococcal sepsis reveals dynamic changes in NK-cell and cytotoxic molecules	Healthy individuals	Patients with meningococcal sepsis (24 hrs after admission)	Pediatrics	Lymphocytes	9	5	4	137.55	162.88	1.18	3.17E-01	3.89E-04	1.18	Affymetrix	Netherlands
		Healthy individuals	Patients with meningococcal sepsis (24 hrs after admission)	Pediatrics	Monocytes	6	3	3	231.49	300.54	1.30	3.83E-01	6.08E-02	1.30		
GSE49754	A Transcriptomic Reporter Assay Employing Neutrophils to Measure Immunogenic Activity of Septic Patients' Plasma (PBMC)	Exposure to plasma from healthy individuals	Exposure to plasma from patients with sepsis	Adults	PBMCs	36	12	24	209.42	246.44	1.18	7.50E-02	4.04E-04	1.18	Illumina	Thailand
GSE49756	A Transcriptomic Reporter Assay Employing Neutrophils to Measure Immunogenic Activity of Septic Patients' Plasma (Expt. 2)	Exposure to plasma from healthy individuals	Exposure to plasma from patients with sepsis	Adults	Neutrophils	46	17	29	11.03	15.03	1.36	1.40E-03	4.10E-03	1.36	Illumina	

Notes:

In vivo: Experimentation or measurements done in whole, living organism or cells without alteration of natural conditions (aside from the collection method).

Ex vivo: Experimentation or measurements done in or on tissue from an organism in an external environment with minimal alteration of natural conditions; e.g. purification of specific cell types.

In vitro: Experimentation or measurements done in or on whole or altered tissue from an organism in an altered external environment; e.g. stimulation of cultured biological specimens.

* If F-test is non-significant ($p < 0.05$), then a two-tailed t-test for equal variance was used.
If F-test is significant ($p < 0.05$), then a two-tailed t-test for unequal variance was used.
A p-value of < 0.05 indicates significant differential expression of ACVR1B between groups.

SI Figure 2

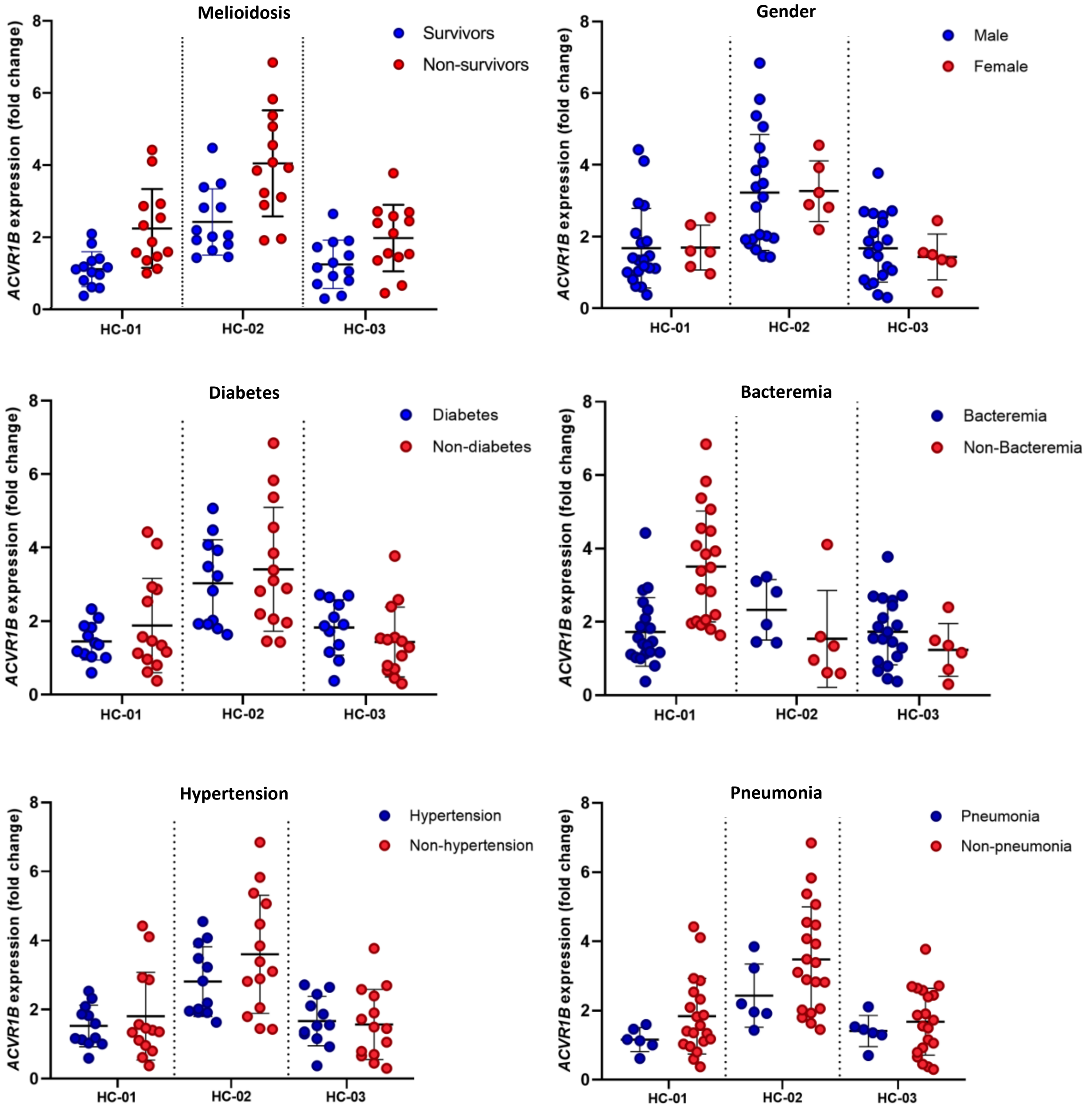

Supplementary Fig 2. Characteristic of melioidosis patients and *ACVR1B* expression. Buffy coat was isolated from heparinized whole blood of three healthy individuals (HC_01, HC_02 and HC_03). Each isolation of buffy coat was exposed to 25% plasma obtained from melioidosis patients (n = 26). *ACVR1B* gene expression of stimulated buffy coat were determined by RT-qPCR. Fold change in *ACVR1B* gene expression of buffy coat responded to melioidosis plasma. Scatter plots of ACVR1B in terms of fold change derived from RT-qPCR analysis across buffy coat exposed to plasma samples. The bar graphs are represented as medians with interquartile ranges and each dot represents data obtained from one individual sample. A Mann-Whitney test was performed for comparison and two-tailed *P* values were calculated.

SI Table 2. Characteristics of healthy donors.

Healthy donors / Characteristics	HC-01	HC-02	HC-03
Ages in years	31	25	31
Gender	Female	Male	Female
Blood type	O	O	AB

Footnotes:
Inclusion criteria: male or female of age ≥18 years.
Exclusion criteria: pregnancy or delivery in the past nine months, weight of less than 40 kg or greater than 136 kg, previous history of melioidosis, recent illness, any chronic medical condition or medications and any organ failure (such as cirrhosis), any immune system deficiency, vaccination within the past six weeks, use of any immune-modifying agents or any anti-inflammatory medications or cell depletion biological agents in the past week, infectious symptoms in the past two weeks, vigorous exercise in the past 24 hours, or alcohol use in the past 24 hours.
